# Computer-assisted surgical anatomical mapping of the medial antebrachial cutaneous nerves. Is there a safe zone for surgery?

**DOI:** 10.1177/17531934261432410

**Published:** 2026-05-27

**Authors:** Lisette C Langenberg, Alexander Poublon, Michiel Zuidam

**Affiliations:** 1 Erasmus Medical Center, Department of Orthopaedic Surgery and Sports Medicine, the Netherlands; 2 Ziekenhuis Gelderse Vallei, Department of Orthopeadic Surgery, the Netherlands; 3 Erasmus Medical Center, Department of Plastic and Reconstructive Surgery, the Netherlands

**Keywords:** Antebrachial nerves, cutaneous nerve anatomy, elbow approaches, medial antebrachial nerve, ulnar nerve surgery

## Abstract

The medial antebrachial cutaneous nerve has a large variation in number and course of its branches. Computer-assisted surgical anatomical mapping of the nerve branches demonstrates no safe zone for surgery around the medial epicondyle of the elbow.

The cutaneous nerves around the elbow are known to have variable courses among individuals (Kwon, 2020). The medial antebrachial cutaneous nerves (MABCN) vary in position, course and branch count around the medial epicondyle. Despite these variations, the position of the MABCN is traditionally estimated by using a set distance compared with anatomical landmarks (Kwon, 2020). Standard distance from local anatomical landmarks may not reliably determine the course of small cutaneous nerves in different age groups and body compositions, such as obese patients. Surgeons could reduce the risk of nerve injury by being shown a nerve-free zone at a distance from anatomical landmarks that can be applied to any patient, regardless of age or body composition. The objective of this study was to show the average course of MABCN branches around the medial epicondyle of the elbow, with an aim of proposing an amendment of medial elbow incisions for portal placement during elbow arthroscopy, in order to decrease the risk of cutaneous nerve injury.

Computer-assisted surgical anatomy mapping allows multiple marked pictures of anatomical specimens to be layered, correcting for three-dimensional differences in body composition, using MagicMorph software (version 1.95; EffectMatrix, Hong Kong). This creates a single illustration of all observed nerve courses that functions like a ‘heat map’ (Langenberg, 2023).

Ten human arm specimens including the hand and the humeral bone fixed with Anubifix embalming solution (developed at the department of anatomy of the Erasmus Medical Center in Rotterdam, the Netherlands) were positioned in neutral forearm rotation and 90° elbow flexion to mimic the position of the elbow during arthroscopy. This same set of specimens was used in a previous study with the elbow in extension to evaluate planning of anterior elbow incisions (Langenberg, 2023). The cutaneous nerves around the elbow were carefully dissected. Anatomical landmarks, visible to the surgeon in the operation room, were marked. These included the medial epicondyle, olecranon tip, ulnar styloid and radial styloid. A grid was made by the placement of pins between these landmarks. A pin was also placed at the traditional position of the proximal anteromedial portal for elbow arthroscopy, which is a standard working portal 2 cm anterior and 2 cm proximal to the medial epicondyle.

The MABCN was found to have multiple branches in most specimens. The anteromedial portal for elbow arthroscopy was very close to the MABCN in all specimens, and dissecting it in 3/10 specimens. A merged image of all MABCN branches revealed a significant variation in its distribution, with no clear zone without cutaneous nerve branches (Figure 1).

**Figure 1.**
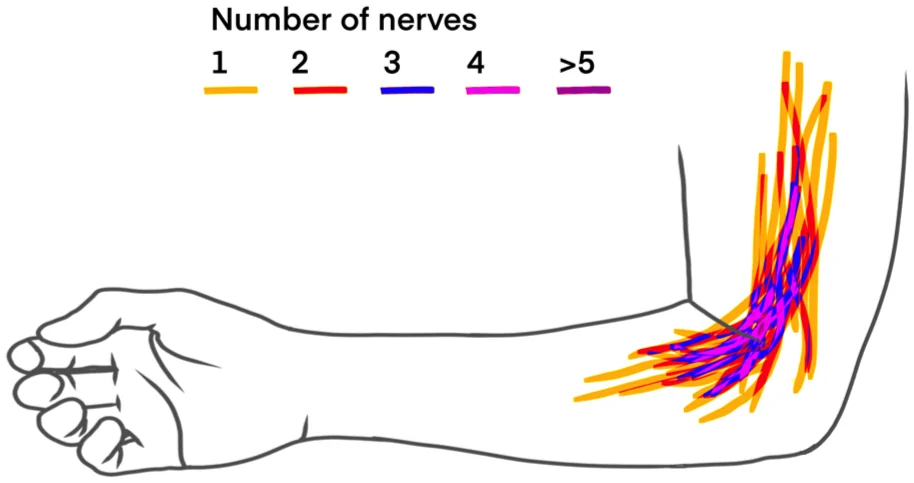
Warped image of medial antebrachial cutaneous nerve of 10 human cadavers overlying each other using computer-assisted surgical anatomical mapping.

There have been previous papers that have suggested modifications to incisions around the medial epicondyle to prevent cutaneous nerve injury. One study that analysed 10 cadavers recommended placing the proximal part of the incision anterior to the medial septum (Kwon, 2020). Advancement of the incision distal to the point where the ulnar nerve enters the flexor carpi ulnaris muscle was previously shown to have a higher risk of MABCN injury (Manoukov, 2020). The computer-assisted surgical anatomy mapping findings suggest that the distal part of the incision should deviate slightly towards the ulna, rather than running parallel to the forearm axis.

Although our study results on the 10 cadaveric specimens are consistent with the previous literature, the sample size remains limited to perform any statistical analysis. Our findings indicating no definable safe zone for the medial approach to the elbow or ulnar nerve are consistent with a study that identified MABCN branches in the surgical wounds of all 97 patients undergoing ulnar nerve decompression (Lowe and Mackinnon, 2004).
